# Monitoring of Cadmium, Lead, and Mercury Levels in Seafood Products: A Ten-Year Analysis

**DOI:** 10.3390/foods14030451

**Published:** 2025-01-30

**Authors:** Luisa Garofalo, Marcello Sala, Claudia Focardi, Patrizio Pasqualetti, Daniela Delfino, Francesca D’Onofrio, Barbara Droghei, Francesca Pasquali, Valentina Nicolini, Flavia Silvia Galli, Paola Scaramozzino, Alessandro Ubaldi, Katia Russo, Bruno Neri

**Affiliations:** 1 Istituto Zooprofilattico Sperimentale del Lazio e della Toscana *M. Aleandri*, Via Appia Nuova 1411, 00178 Roma, Italy; marcello.sala@izslt.it (M.S.); daniela.delfino@izslt.it (D.D.); francesca.donofrio@izslt.it (F.D.); barbara.droghei-esterno@izslt.it (B.D.); francesca.pasquali-esterno@izslt.it (F.P.); valentina.nicolini-esterno@izslt.it (V.N.); flavia.galli@izslt.it (F.S.G.); paola.scaramozzino@izslt.it (P.S.); alessandro.ubaldi@izslt.it (A.U.); katia.russo@izslt.it (K.R.); bruno.neri@izslt.it (B.N.); 2 Istituto Zooprofilattico Sperimentale del Lazio e della Toscana *M. Aleandri*, Via di Castelpulci, San Martino alla Palma, 50010 Firenze, Italy; claudia.focardi@izslt.it; 3 Dipartimento di Sanità Pubblica e Malattie Infettive, Sapienza Università di Roma, Piazzale Aldo Moro 5, 00185 Roma, Italy; patrizio.pasqualetti@uniroma1.it

**Keywords:** heavy metals, contaminants, CE regulation, fishery products, official controls, chemical tests, quantification, toxicity, frequent consumers

## Abstract

The European Food Safety Authority (EFSA) monitors the presence and concentration of contaminants in food to mitigate health risks. EU legislation sets maximum levels of heavy metals in foods, including cadmium (Cd), lead (Pb), and total Hg (THg) in seafood, due to their toxicity. In the framework of official control, between 2014 and 2023, 5854 seafood samples were collected and 4300 analyses for THg, 3338 for Cd, and 2171 for Pb were performed using Inductively Coupled Plasma Mass Spectrometry (ICP-MS) and cold vapor atomic absorption spectrometry (CVAAS). The aim was to assess the proportion of contaminated foods in the dataset, the concentration of contaminants, and the potential health risks associated with their intake. Of the total samples analyzed, 142 (2.43%) were found to be non-compliant (n.c.). Concentrations exceeding the limits for Cd were primarily detected in cephalopods (n = 17), mainly squids. In contrast, Hg levels exceeded the limits in marine fish (n = 118), notably in swordfish (11.30% of n.c. samples among those analyzed for this species), sharks (6.48%), and tuna species (3.11%). Regarding Pb, only a single bivalve sample was found to exceed the maximum limits. A preliminary assessment of weekly exposure to Hg through swordfish consumption raised concerns about the frequent intake of marine top predators, particularly for vulnerable people.

## 1. Introduction

Fish consumption is widely recognized as an essential component of a balanced diet due to its provision of important nutrients, such as high-quality proteins, vitamins, minerals, and polyunsaturated fatty acids with cardioprotective properties [1]. However, the marine environment has become increasingly polluted by human activities, with significant amounts of chemicals entering aquatic systems. Among these pollutants, heavy metals are of particular concern due to their persistence and high toxicity, even at very low concentrations [2]. Heavy metals in the environment originate from both natural and anthropogenic sources. Marine pollution primarily results from contaminants transported by rivers, which pass through areas of industrial agricultural activities, or are directly released into coastal waters through urban and industrial wastewaters. It is estimated that at least half of marine cadmium (Cd) contamination originates from anthropogenic activities, such as waste from the production of zinc, plastic, batteries, and paints. Cadmium is also released by waste incinerators and is present in phosphate fertilizers. This element is easily absorbed by marine organisms but is difficult to eliminate. It transfers from sediments and tends to concentrate in phytoplankton and macrophytes, subsequently bioaccumulating in crustaceans, echinoderms, and mollusks (e.g., bivalves, gastropods, and cephalopods). Benthic organisms, thus, represent effective bioindicators for monitoring pollution in marine environments caused by this and other heavy metals [3,4,5]. In fish, Cd concentrations are usually lower, accumulating mainly in the kidneys and tissues such as the gills and hepatopancreas [6].

Lead (Pb) is a naturally occurring metal released into the atmosphere by the enamel and paint industries during steel melting processes and from the combustion of fossil fuels, including gasoline (until its removal). Pb is also introduced into aquatic environments through surface runoff from soil and atmospheric deposition. Its concentrations in the sea are primarily regulated by absorption into sediments or particulate matter. Fortunately, dietary intake of lead has decreased in recent years due to global preventive measures.

Mercury (Hg) contamination in the environment originates from both natural and anthropogenic sources. The pulp and paper industries and chlor-alkali plants are among the most hazardous for Hg emissions. Although the industrial use of mercury has significantly decreased in recent years due to concerns over its impact on the food chain, its presence in the environment remains persistent, mainly because of its stability in atmospheric precipitation and sediments. In marine sediments, mercury is converted by microorganisms into its organic form, methylmercury (MeHg), which is extremely toxic. This metal bioaccumulates and biomagnifies along the food chain, resulting in higher concentrations in predatory fish, primarily in their muscle tissues. The proportion of organic and inorganic mercury varies by animal species, age, size, and water pH; however, MeHg is the predominant form found in fish and other seafood [7].

Heavy metals can have toxic effects on kidney (Pb, Cd, Hg) and liver (Pb, Cd) function, reduce cognitive (Pb, Hg) and reproductive (Cd, Pb) abilities, cause hypertension (Cd), and lead to neurological problems (Hg, Pb). In some cases, these metals may have carcinogenic effects (Cd) [8]. The consumption of foods with high levels of MeHg is especially dangerous for pregnant women due to its teratogenic effects, and for children during neurodevelopment [9]. Consequently, the levels of heavy metal contamination in fish products must be carefully monitored to minimize health risks.

Risk assessments are carried out by the European Food Safety Agency (EFSA) to evaluate consumer exposure to various food contaminants and establish maximum limits for potentially toxic substances. The EFSA’s comprehensive assessment relies on data collected through monitoring activities conducted by EU Member States. The *Istituto Zooprofilattico Sperimentale del Lazio e della Toscana* (IZSLT), like the other regional IIZZSS, actively contributes to ensuring compliance with legal limits for food products submitted by competent authorities and private companies. The IZSLT also supports national and regional control planning. Chemical essays to assess levels of Cd, Pb, and Hg, regulated by Commission Regulation (EU) 2023/915 [10], which repealed Regulation (EC) No 1881/2006 [11], are usually requested as part of official controls. These include inspections conducted at Border Control Posts (BCPs), Maritime, Air, and Border Health Offices (USMAFs), and Veterinary Offices for the Fulfillment of Community Obligations (UVACs). Additional controls are carried out within the framework of Official Plans for Residual Contaminants and by local health services (ASL) in warehouses, markets, fish processing plants, wholesale and retail outlets, shellfish purification centers, public or collective catering establishments, boats, or other vehicles.

This study, based on the diagnostic activity of IZSLT over a ten-year period (2014–2023), focused on detecting Cd, Pb, and Hg in seafood. Compliance was assessed relative to maximum limits established by EU Regulations. The aims of this work were to 1. analyze the concentrations of the three heavy metals regulated at the European level, considering the taxonomic groups of the samples; 2. identify non-compliant samples and specific patterns of irregularities; and 3. assess the potential health risks associated with excessive seafood consumption, particularly for species with the highest contamination levels.

## 2. Materials and Methods

### 2.1. Samples and Chemical Analyses

**Figure 1:**
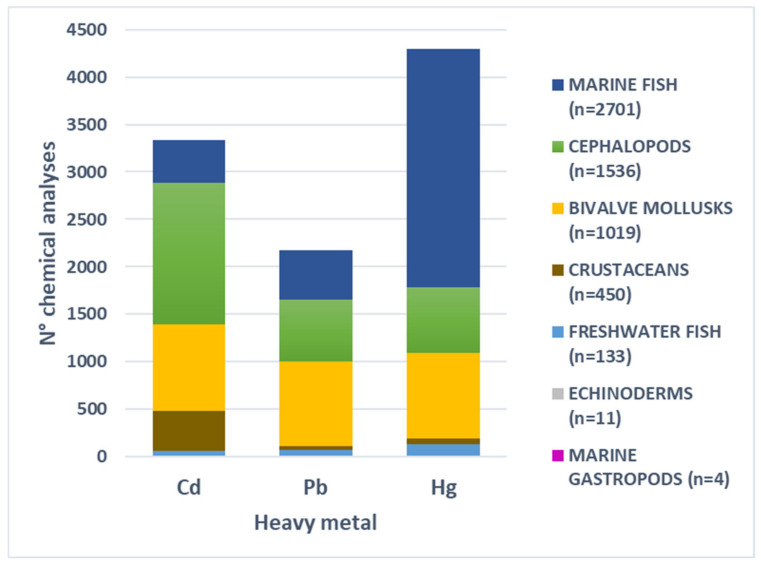
Number of chemical analyses conducted for the three heavy metals across various taxonomic groups of seafood. The number of samples analyzed for each taxonomic group is indicated in parentheses within the legend.

Between 2014 and 2023, IZSLT analyzed a total of 5854 seafood samples from around the world for heavy metal contamination. These samples represented seven taxonomic groups: marine fish (46.14%), cephalopod mollusks (26.24%), bivalve mollusks (17.41%), crustaceans (7.69%), freshwater fish (2.27%), and a very small proportion (<1%) of echinoderms and marine gastropods (Figure 1). For further analyses and categorization, the requests for contaminant testing were grouped into four sampling plan types: i. the Hazard Analysis and Critical Control Points (HACCP) self-control plan for food companies; ii. official import control; iii. official control of food during processing; and iv. trade and official control of Italian primary production. Upon collection, the samples were placed into sterile bags, transported to the laboratory, and stored at −20 °C until analysis. In total, 9809 chemical analyses were conducted by the IZSLT chemical laboratories in Rome and Florence. These included 4300 tests for total Hg (THg), 3338 for Cd, and 2171 for Pb (Figure 1).

**Figure 2:**
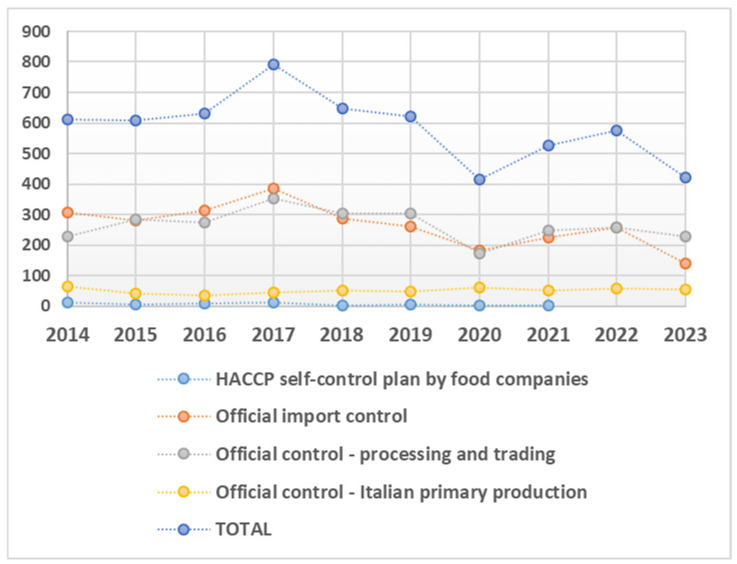
Number of chemical analyses conducted over different years, categorized by the type of sampling plan.

The analyses were distributed across the ten years of the study period and categorized according to the four types of sampling plans, as shown in Figure 2.

### 2.2. Analytical Procedure

All chemicals and reagents used were of analytical grade. Nitric acid (67–69%), hydrogen peroxide (30–32%), and hydrochloric acid (34–37%) for trace analysis were supplied by Carlo ERBA Reagents (Cornaredo, MI, Italy). High-purity water (resistivity > 18 MΩ) was obtained from a Milli-Q purification system Arium Pro Ultrapure Lab Water Systems, provided by Sartorius Italy Srl (Varedo, MB, Italy). Multi-element certified reference material (1 mg/L), Hg-certified reference material, and certified solutions of Germanium (Ge), Yttrium (Y), and Indium (In), at a concentration of (1000 mg/L), were sourced from CPAChem (Stara Zagora, Bulgaria).

Prior to analysis, Teflon digestion tubes were washed with nitric acid and thoroughly rinsed with Milli-Q water to prevent contamination. A 1.0 g aliquot of each homogenized sample was weighed into the tubes, followed by the addition of 8.0 mL of HNO_3_ (67–69%) and 1.0 mL of H_2_O_2_. Digestion was performed in a MILESTONE ETHOS ONE SK10T microwave oven (Milestone, Shelton, CT, USA) at 1500 W and 190 °C for 20 min. After cooling, the digested contents were transferred to decontaminated volumetric flasks, diluted to 100 mL with Milli-Q water, and analyzed. Reagent blanks were prepared under the same conditions. An internal standard mixture solution containing Ge, In, and Y was added to all samples, blanks, and calibration standards at a final concentration of 1 µg/mL.

During the study period, several parameters and equipment were employed, but the description here reflects the methodologies currently in use. Analyses were conducted using an inductively coupled plasma mass spectrometer (ICP-MS) Thermo Fisher Icap Q (Thermo Fisher Scientific, Waltham, MA, USA) equipped with a CETAC 500 Series ASX-520 Auto (CETAC Technologies, Omaha, NE, USA). The primary operating parameters were radio frequency power 1550 W, auxiliary gas flow 0.8 L/min, nebulizer flow 1.06 L/min, and plasma gas flow 14.0 L/min. Daily tuning of the instrument ensured optimal signal stability, sensitivity, and minimal interference effects.

The sampling and validation of the analytical method were performed according to the requirements of Commission Regulation (EC) N° 333/2007. The method was evaluated for specificity, linearity, limit of detection (LOD), limit of quantification (LOQ), repeatability, reproducibility, recovery, and precision.

Calibration standards for the metals were prepared at concentrations of 0.1, 0.5, 1.0, 5.0, and 20.0 ng/mL for Cd and Pb and 0.05, 0.1, 0.5, 1.0, and 2.0 ng/mL for Hg. Isotopes used were ^111^Cd, ^206,207,208^Pb, and ^200^Hg. LODs and LOQs were established at 0.006 mg/kg and 0.020 mg/kg for Cd and Pb and 0.010 mg/kg and 0.030 mg/kg for Hg, respectively. Linearity was confirmed with the correlation coefficient (R^2^) exceeding 0.999. Each measurement was performed in triplicate, and values were reported as milligrams per kilogram (mg/kg) on wet weight basis, in accordance with European regulations. Quality control was ensured using DORM-5 reference material (National Research Council Canada), which was analyzed in duplicate during each analytical session and processed identically to the samples. Recovery values between 80% and 120% were considered acceptable; if the values fell outside this range, the analytical session was repeated.

Total mercury was also determined using cold vapor atomic absorption spectrometry (CVAAS) with a Flow Injection Mercury System (FIMS-100, PerkinElmer, Waltham, MA, USA) equipped with an AS-90 autosampler. A 0.2% (*w*/*v*) sodium borohydride solution (Thermo Fisher Scientific, Waltham, MA, USA) in 0.05% (*w*/*v*) sodium hydroxide (Sigma-Aldrich, MO, USA) served as the reducing agent, while a 3% (*v*/*v*) hydrochloric acid solution was used as the carrier. Calibration standards for Hg concentrations were prepared at concentrations of 2.0, 10.0, 20.0, 40.0, and 50.0 ng/mL and by diluting stock solutions (1 mg/L) in 3% *v*/*v* HCl. In cases where the concentration of heavy metals in a sample approached or exceeded 80% of the maximum limit established by EU regulations, the sample was analyzed in duplicate. The compliance result was assessed considering the measurement uncertainty.

### 2.3. Maximum Limits for the Analyzed Seafood

Maximum levels (MLs) for the three heavy metals in different seafood products are established by Commission Regulation (EU) 2023/915, which repealed Regulation (EC) No 1881/2006. Although subsequent amendments to the regulation have been made, the MLs for the analytes within the taxonomic groups examined here have remained unchanged over the years (Table 1). The MLs for Cd and Hg increase with the trophic level of the marine fish species under consideration, with higher thresholds designated for predatory fish. For echinoderms, based on guidelines provided by the Italian Ministry of Health, the limits applied are equivalent to those for bivalves (Cd and Pb) and non-predatory fish (Hg).

### 2.4. Statistical Analysis

Statistical analyses to describe the dataset and assess concentrations of each analyte were performed using Stata/SE 16.1 (StataCorp LP, College Station, TX, USA). For each taxonomic group–heavy metal combination, the following descriptive statistics were calculated: mean, standard deviation, median, maximum value, 95th percentile (p95), and 99th percentile (p99). Values of 0.001 and 0 were used for data analyses when results were <LOQ and <LOD, respectively. Box plots were generated to explore the concentrations of Cd and THg for non-compliant samples, focusing on the major species groups, and descriptive statistics were additionally provided for the marine fish with THg concentrations above the legal limits, detailing results for five uniform groups of species/maximum limit of THg.

### 2.5. Dietary Intake Calculation

The EFSA has established Tolerable Weekly Intakes (TWIs), or “safe levels”, which estimate the maximum amount of a potentially harmful substance or contaminant (e.g., heavy metals) that can be ingested weekly per unit body weight without adverse health effects. The EFSA has also provided dietary recommendations for the most vulnerable groups, on a geo-referenced basis, and shared them with European Union National Authorities [7]. The EFSA Comprehensive European Food Consumption Database (https://www.efsa.europa.eu/en/microstrategy/food-consumption-survey, accessed on 4 October 2024) was checked to obtain Italian consumption data for the fish species which would have resulted in the highest mercury levels in our study. Estimated Weekly Intakes (EWIs) were calculated for them according to methods explained in the previous literature [12,13].

## 3. Results

### 3.1. Levels of Heavy Metals in the Analyzed Seafood

**Figure 3:**
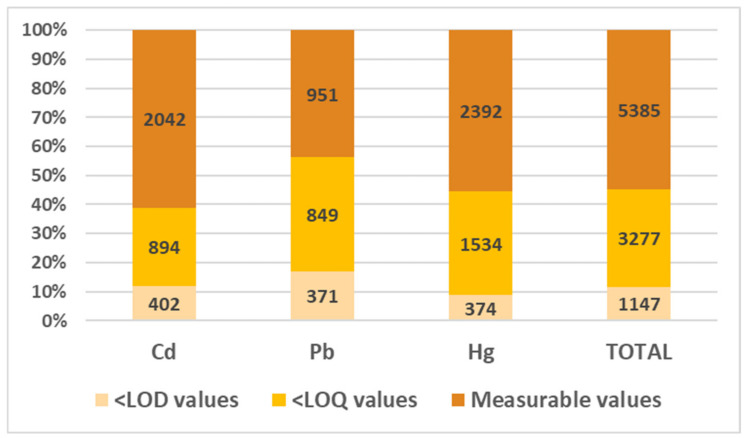
Percentage distribution of the three outcomes for the n = 9809 chemical analyses carried out for each heavy metal. Outcomes include the following: below the limit of detection (LOD), below the limit of quantification (LOQ), and measurable values. Note: LODs and LOQs were 0.006 mg/kg and 0.020 mg/kg for Cd and Pb and 0.010 mg/kg and 0.030 mg/kg for Hg, respectively.

The analyses performed to detect the three analytes (n = 9809) were fairly evenly distributed across the years (Figure 3), with a slight increase in 2017 attributable to adjustments in the national sampling plans and an minor decline in 2020 due to movement and commercial restrictions associated with the COVID-19 pandemic. Chemical detections revealed that 55% were measurable values (n = 5385), while 33% were below the limit of quantification (<LOQ; n = 3277) and 12% fell below the limit of detection (<LOD; n = 1147). As detailed in Figure 3, the analysis for Pb yielded the lowest percentage of measurable values (44%) compared to 56% for Hg and 61% for Cd. Conversely, lead had the highest percentage of <LOQ (39% compared to 36% for Hg and 27% for Cd) and the highest percentage of <LOD values (17% compared to 9% for Hg and 12% for Cd).

#### 3.1.1. Cadmium

Mollusks were the primary group of seafood tested for Cd, with n = 1500 cephalopods, n = 904 bivalves, and n = 2 marine gastropods. Table 2 summarizes the key parameters of cadmium concentration obtained from the n = 3338 analyses carried out. Notably, cephalopods showed the highest mean Cd concentration (0.15 ± 0.38 mg/kg) and the highest absolute value detected (5.60 mg/kg, identified in an oceanic squid). In addition, cephalopods showed higher p95 and p99 values compared to the other groups. Bivalves, crustaceans, and the small sample of echinoderms also presented cadmium levels higher than those found in marine and freshwater fish.

#### 3.1.2. Lead

Mollusks, including n = 650 cephalopods, n = 897 bivalves, and n = 4 marine gastropods, constituted the largest group of seafood analyzed for Pb. Table 3 presents the main parameters for lead concentration obtained from the 2171 chemical analyses carried out. Among the analyzed samples, bivalves exhibited the highest mean Pb concentrations (0.13 ± 0.16 mg/kg) and the highest absolute value (3.20 mg/kg, found in a truncate donax). Additionally, bivalves showed higher p95 and p99 values compared to other groups. Notable Pb concentrations were also observed in echinoderms. However, due to the limited sample size (n = 11), additional sampling and analysis are recommended to better assess the contamination in this group. Fish samples consistently exhibited low Pb concentrations, with mean and median values always below LOD or LOD.

#### 3.1.3. Total Mercury

Out of the 4300 chemical analyses to detect total mercury (THg), marine fish constituted the largest group of seafood analyzed (n = 2519). This taxonomic group exhibited the highest mean value (0.39 ± 0.51 mg/kg) and the highest absolute value (13.00 mg/kg, found in a marlin) among all the samples analyzed (Table 4). Furthermore, marine fish displayed a 95th percentile and 99th percentile of 1.20 mg/kg and 1.90 mg/kg, respectively. Freshwater fish, in contrast, showed lower concentrations of mercury, always within the established limits. According to the Mann–Whitney test, the concentration of mercury in freshwater fish was significantly lower than in marine fish (U = 255,206.5, U-derived Z = 9.348, *p* < 0.001).

### 3.2. Non-Compliant Samples

Across the entire dataset, 142 samples (2.43% of those analyzed) were found to be non-compliant (n.c.) with EU regulations. Most of these samples were collected during official inspections of food in commercial phases (e.g., warehouses and fish markets) by local health authorities (n = 102), or during import controls (n = 36) conducted by border control agents at ports (Table 5). Notably, no freshwater fish, echinoderm, or marine gastropods had heavy metal concentrations exceeding the regulatory limits established for their respective groups.

Cadmium concentrations above the legal limits were predominantly found in cephalopod mollusks (n = 17), while mercury levels exceeding permitted limits were primarily observed in marine fish (n = 118). Moreover, sporadic cases of non-compliant taxon–analyte combinations were identified (Table 6). Specifically, three Chilean mussels (*Mytilus chilensis*) and two American lobsters (*Homarus americanus*) were found to exceed EU limits for [Cd]. Furthermore, a squid from Peru exhibited a [Hg] concentration of 1.90 mg/kg, while a sample of truncate donax (*Donax trunculus*) from Lazio, Italy, had a [Pb] level of 3.20 mg/kg. In this particular sample, other contaminants were also determined, including Arsenic (at a concentration of 2.5 mg/kg); the sum of the concentration of the six indicator non-dioxin-like polychlorinated biphenyls (ndl-PCBs) was 1.53 ng/g, the value of WHO-PCDD/PCDF-TEQ/g (upper bound) was 0.0574 pg-TEQ-WHO/g wet weight, and the value of WHO-PCDD/PCDF-TEQ/g (upper bound) was 0.175 pg-TEQ-WHO/g wet weight.

**Figure 4:**
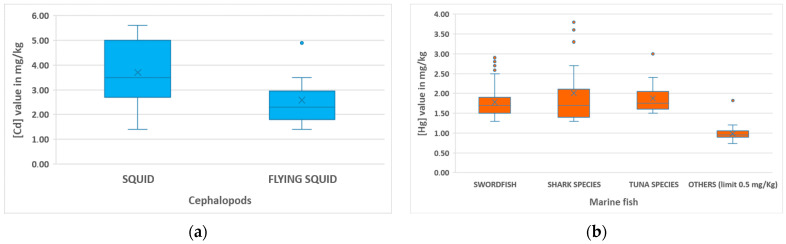
Box plots of (**a**) [Cd] (mg/kg) values of non-compliant samples of the two main cephalopods groups (squid and flying squid). Data for octopus and cuttlefish are not represented. (**b**) Total [Hg] (mg/kg) values of non-compliant samples for each group of marine fish species (swordfish: n = 59; shark species: n = 16; tuna species: n = 14; others with EU limit of 0.5 mg/kg: n = 26). Data from other species (n = 3) with a [Hg] limit of 1.0 mg/kg are not plotted. The median value is represented by a horizontal line, while X indicates the mean value.

The 17 cephalopods exceeding the EU limit of 1.00 mg/kg for cadmium exhibited concentrations ranging from 1.40 to 5.60 mg/kg, with a mean value of 2.96 ± 1.46 mg/kg. These were mainly squid (n = 7) and flying squid (n = 7; Figure 4a), as well as one cuttlefish and two octopuses.

The marine fish with total mercury concentrations above the legal limits (Table 7 and Figure 4b) were primarily swordfish (*Xiphias gladius)* (n = 59, representing 11.30% of non-compliant samples for this species analyte; [Hg] range: 1.30–2.90 mg/kg, mean 1.78 ± 0.38 mg/kg), shark species (n = 16, accounting for 6.48% of non-compliant samples in this group analyte; [Hg] range: 1.30–3.80 mg/kg, mean 2.01 ± 0.85 mg/kg), and tuna species (n = 14, corresponding to 3.11% of non-compliant samples in this group analyte; [Hg] range: 1.50–3.00 mg/kg, mean 1.88 ± 0.41 mg/kg). Additionally, a Pacific marlin, another marine top predator, had an extremely high [Hg] value of 13.00 mg/kg, far exceeding the legal limit of 1.0 mg/kg limit. Furthermore, 26 fish samples of different species not listed above exceeded their specific [Hg] limit of 0.5 mg/kg.

### 3.3. Pilot Evaluation of EWI for Swordfish

Italian consumption data for the fish species with the highest mercury levels found in our study (Table 7) were derived from two surveys [14,15] within the EFSA Comprehensive European Food Consumption Database. The mean and median values of total mercury concentrations here obtained were used to calculate EWI under two scenarios: one based on mean fish consumption for all subjects interviewed and another for age categories where more than 10 individuals were reported as consumers. Three out of ten population categories considered in the two surveys [14,15] showed more than ten consumers of swordfish and their mean consumption per week, along with EWI of THg, as reported in Table 8.

Conversely, EWI was not calculated for sharks (i.e., the taxonomic group that has mean and median values of THg comparable to those found for swordfish) because of two reasons: the first is that information for shark consumption in these two survey was limited to “smooth hounds” (*Mustelus* species). The term “sharks” indeed refers to all members of the class Chondrichthyes, which includes approximately 530–540 species, among which *Mustelus* sp., *Prionace* sp., *Isurus* sp., and *Squalus* sp. are those consumed as food in Italy, and our dataset encompassed a broader range of shark species than the *Mustelus* ones. The second is that fewer than 10 individuals per age category reported consuming this seafood category, making it unsuitable for robust data analysis. Therefore, our exploratory evaluation of EWI focused solely on swordfish, as species-specific consumption data for this category was explicitly reported in both surveys and a greater number of people were reported to consume this species.

In line with the Joint FAO/WHO Expert Committee on Food Additives (JECFA), the EFSA CONTAM Panel in 2012 established a revised tolerable weekly intake (TWI) of 4 µg/kg b.w. for inorganic mercury (iHg) and of 1.3 µg/kg b.w. for methylmercury (MeHg). Previous studies found a MeHg/THg ratio of 73% in swordfish from the Mediterranean area [12]. However, EFSA’s conservative approach suggests that the occurrence of MeHg in predatory fish can be considered equivalent to 100% of the THg average concentration [7].

Based on our estimates (Table 8), the EWI of total mercury ingestion through swordfish alone, calculated using the mean and median concentrations found in this work, would fall within the TWI for both iHg and MeHg in the general population. However, frequent fish consumers might exceed the TWI thresholds.

## 4. Discussion

This work presents a survey on a convenience sample of seafood products derived from a decade of official controls and surveillance in food distribution and production centers in central Italy. Nearly 10,000 chemical analyses were performed on products from all over the world and belonging to six taxonomic groups to assess their compliance with European regulations on heavy metal concentrations.

A total of 2.43% of the analyzed samples were found to be non-compliant with EU regulations. The majority of these cases involved total mercury and cadmium concentrations, with only one sample exceeding the maximum level set for lead.

Bivalve mollusks were the taxonomic group with the highest relative concentration of lead in our dataset. However, our analysis confirmed that overall levels of this metal in the aquatic environment, as represented by a mixed seafood sample, do not appear to be alarming. This is probably because our sampling did not include known active contamination hotspots such as the Taranto Gulf (Italy) [4]; western Norway [16]; East China Sea [17]; or other global hotspots [18]. Cephalopods exhibited the highest cadmium concentration, with 17 samples exceeding the EU regulatory limit for human consumption. This pattern is well documented in the literature [19], above all for oceanic squid, which can reach large sizes and occupy high trophic levels in the marine food web [20]. These organisms can transfer contaminants to their predators (i.e., cetaceans and sea turtles) as well as to humans. A study conducted on seafood from Spanish markets [21] found that 15% of the analyzed cephalopods were non-compliant with EU regulations. Additionally, a survey using molecular barcoding at the Border Inspection Post of Livorno-Pisa (Italy) identified this taxonomic group as having the highest mislabeling rates among fishery products [22]. This mislabeling is often unintentional in cephalopods as squids, flying squids, and cuttlefish can be easily confused morphologically. However, it can also be intentional; for instance, selling a lesser-known species under the name of a highly commercial and depleted species may confer economic advantages. [22]. For other taxonomic groups, such as marine fish, mislabeling may lead to the incorrect application of contaminant limits to the wrong species. Therefore, we recommend implementing stricter controls to ensure compliance with heavy metal limits and accurate labeling.

Chilean blue mussels (*Mytilus chilensis*) were found, here, to contain cadmium above the permissible limit, confirming their role as bioaccumulators of contaminants along the Chilean coast [23] due to their sedentary filter-feeder habits.

Similarly, contamination has been reported in some North American coastal waters, from which two American lobsters (*Homarus americanus*) in our dataset, non-compliant for cadmium, were sourced. In these environments, crustaceans often suffer from secondary infections and immune system deficiency due to heavy metal accumulation [24], making them effective bioindicators of ecosystem health.

Tracing the origin of seafood products is, therefore, essential not only for labeling the exporting country, but also the specific sampling area (i.e., FAO fishing area), particularly for sessile organisms linked to narrow geographical regions. Some countries may in fact display some regions with higher pollution than others, and contain different FAO fishing zones within the same country. Consequently, official controls and diagnostic analyses in importing countries are essential to prevent contaminated food from reaching consumers.

Regarding mercury concentrations, marine fish exhibited the highest levels of this element in our dataset, consistent with findings from similar studies [21,25]. An exception was found in a single squid from Peru, which had a Hg level exceeding the EU limits, likely reflecting historical contamination caused by gold mining practices [26].

Freshwater fish, mainly African and Asiatic species in our dataset, did not exceed EU mercury limits. This is comforting from of a food safety perspective, as species such as pangasius and tilapia are often included in school menus and public food services. However, ongoing monitoring is advised, especially since certain populations in Africa and Asia rely heavily on single fish species for their diets [27,28].

Within marine fish, pelagic species exhibited the highest total mercury concentrations, consistent with their apex trophic positions in the food web. In our dataset, 11.30% of swordfish, 6.48% of sharks, and 3.11% of tuna samples exceeded EU regulatory limits for total Hg. These findings are consistent with previous studies conducted in other Italian regions, which reported varying non-compliance rates (e.g., 17.30% for swordfish in southern Italy [29], 7.69% in northern Italy [25]). Much higher percentages of illegal swordfish were reported in other countries: 37% in Spain [30], 80% in Canada [31], 67% in the United States [32], and 67% in Sri Lanka [33]. Notably, tuna and swordfish are among the most commonly consumed fish species in Italy, particularly in coastal areas [13], which highlights the potential risk of mercury exposure for local consumers. In contrast, shark consumption is less common in Italy but more frequent worldwide, with significant non-compliance rates reported in Spain, Canada, and Korea [21,31,34]. Other large marine predators should also be monitored for contaminant levels, as highlighted by the Pacific marlin sample in our dataset, which had a THg concentration of 13.00 mg/kg. This level is comparable to the concentration of 12.70 mg/kg found in marlin muscles from the Gulf of California and the Gulf of Mexico [35,36]. These results highlight the importance of monitoring mercury levels in various fish as emphasized by EC Recommendation 2022/1342 [37]. According to the IUCN Red List of Threatened Species (2024) [38], several top predator species including sharks, swordfish, and billfish are currently showing a decreasing population trend worldwide: one more reason to limit their capture for food.

Finally, farmed fish and shellfish from Italy (i.e., the samples with the official control, Italian primary production request in Table 5), comprising almost 500 samples in this survey, demonstrated low contamination levels, with only one non-compliance detected. This finding is consistent with the literature suggesting that farmed seafood generally has lower contamination levels [39,40].

When compared to similar studies in Italy [25,41] and Europe [21,42], our findings confirm a general pattern of compliance with European legislation. However, our preliminary risk assessment raised concerns about chronic mercury intake in frequent consumers of marine top predators. The data collected in this study on heavy metal levels in seafood products may be useful for ongoing monitoring efforts and provide valuable estimates of heavy metal intake from this type of food matrix.

Future evaluations should include additional food items and assess acute risks for vulnerable populations, including children and the elderly, who may be disproportionately affected by the occasional consumption of highly contaminated samples. Pregnant women are also at high risk, with mercury levels in fetal blood that can be about 1.7 times higher than those recorded in maternal blood [43] and can bring teratogenic effects on fetus development if consumed in delicate phases of pregnancy.

A general recommendation is to prioritize the consumption of herbivorous or small predatory species (e.g., red mullet, plaice, mackerel, salmon, sea bream, and sea bass), which have lower methylmercury levels compared to apex predators (40–60% of THg compared to almost 100%, respectively [42]), limiting consumption of the latter to a few portions/week. Furthermore, strategies such as dietary diversification, improved traceability of the products, and consumer education are pivotal in reducing the health risks associated with heavy metal intake.

For the other two metals considered here, it has to be noticed that other types of foods (e.g., chocolate, cereal-based products) also contribute to a significant intake of these heavy metals [44] and should be taken into account when assessing the tolerable intake for the organism. Finally, it should be remembered that smoking, chronic diseases (e.g., diabetes and hypertension), and nutritional factors (e.g., iron deficiency) can also influence the body’s response to high levels of contaminants.

## 5. Conclusions

This study, carried out over a ten-year period by the IZSLT in collaboration with other Italian public health and veterinary authorities, provides valuable information on the levels of three heavy metals found in seafood products commonly available in central Italian markets. The results showed that a limited percentage of samples (2.43%) did not comply with European regulations, primarily due to mercury and cadmium concentrations. Cadmium levels were particularly high in cephalopods, while mercury levels were significantly elevated in marine fish, especially in top predators such as swordfish, sharks, and tuna. Previously identified world hotspots of heavy metal contamination were confirmed, with sporadic occurrences observed in shellfish and crustaceans within our dataset. In addition, well-known patterns of increasing contamination along the marine trophic chain were observed. Despite the relatively low incidence of non-compliance, the results underline the importance of continuous monitoring, especially for fish products that pose the highest risks. It is also important to consider that other foods contribute to contaminant exposure and must be accounted for in food safety assessments. These findings, if effectively communicated and disseminated to consumers, could lead to greater awareness when purchasing food products, especially for high-level fish consumers and more vulnerable population groups.

## Figures and Tables

**Table 1:** Maximum levels (mg/kg) for the three heavy metals in the taxonomic groups considered, according to the Commission Regulation (EU) 2023/915. Different limits for marine fish depend on the species and are specified in the Supplementary Materials.

*EU Maximum* Limits (MLs in mg/kg)			
Taxonomic Group	Cd	Pb	THg
Marine fish	0.05, 0.10, 0.15, 0.25	0.30	0.30, 0.50, 1.00
Cephalopods	1.00	0.30	0.30
Bivalve mollusks	1.00	1.50	0.50
Crustaceans	0.50	0.50	0.50
Freshwater fish	0.05	0.30	0.50
Echinoderms	1.00	1.50	0.50
Marine gastropods	1.00	0.30	0.30

**Table 2:** Main parameters of cadmium concentration in different taxonomic groups analyzed. Concentration values are expressed in mg/kg.

Cadmium						
Taxonomic Group	n	Mean ± sd	Median	Max	p95	p99
Marine fish	450	<LOQ	<LOD	0.90	0.05	0.20
Cephalopods	1500	0.15 ± 0.38	0.05	5.60	0.60	1.45
Bivalve mollusks	904	0.08 ± 0.14	0.06	1.60	0.27	0.70
Crustaceans	424	0.08 ± 0.13	0.03	0.85	0.33	0.60
Freshwater fish	53	<LOD	<LOD	0.04	<LOD	0.04
Echinoderms	5	0.06 ± 0.04	0.07	0.09	0.09	0.09
Marine gastropods	2	/	/	0.78	/	/
**Total**	**3338**	**0.10 ± 0.28**	**0.04**	**5.60**	**0.41**	**1.00**

**Table 3:** Main parameters of lead concentration across taxonomic groups. Concentration values are expressed in mg/kg.

Lead						
Taxonomic Group	n	Mean ± sd	Median	Max	p95	p99
Marine fish	514	<LOQ	<LOD	0.22	0.04	0.12
Cephalopods	650	<LOQ	<LOD	0.27	0.06	0.17
Bivalve mollusks	897	0.13 ± 0.16	0.11	3.20	0.40	0.57
Crustaceans	38	0.02 ± 0.06	<LOD	0.34	0.13	0.34
Freshwater fish	57	<LOD	<LOD	0.04	0.03	0.04
Echinoderms	11	0.39 ± 0.42	0.41	1.20	1.20	1.20
Marine gastropods	4	/	/	0.34	/	/
**Total**	**2171**	**0.06 ± 0.13**	<LOD	**3.20**	**0.26**	**0.49**

**Table 4:** Main parameters of total mercury concentration obtained for different taxonomic groups analyzed. Concentration values are expressed in mg/kg.

Total Mercury						
Taxonomic Groups	n	Mean ± sd	Median	Max	p95	p99
Marine fish	2519	0.39 ± 0.51	0.23	13.00	1.20	1.90
Cephalopods	687	0.02 ± 0.09	<LOD	1.90	0.11	0.24
Bivalve mollusks	898	0.01 ± 0.03	<LOD	0.47	0.04	0.14
Crustaceans	60	0.10 ± 0.12	0.06	0.52	0.39	0.52
Freshwater fish	123	0.07 ± 0.09	0.05	0.65	0.23	0.36
Echinoderms	11	<LOQ	<LOD	0.05	0.05	0.05
Marine gastropods	2	/	/	0.32	/	/
**Total**	**4300**	**0.24 ± 0.43**	**0.04**	**13.00**	**1.00**	**1.75**

**Table 5:** Number of non-compliant (n.c.) samples for each type of sampling/analysis request. In parentheses, the proportion of n.c. samples relative to the total for each taxonomic group–request combination.

n.c. per Sampling Request	Marine Fish	Cephalopods	Bivalve Mollusks	Crustaceans	Total
HACCP self-control plan by food companies	3 (9.09%)				**3 (6.25%)**
*n analyzed*	*33*	*3*	*11*	*1*	***48***
Official import control	24 (2.05%)	7 (0.70%)	3 (8.57%)	2 (0.61%)	**36 (1.42%)**
*n analyzed*	*1169*	*999*	*35*	*325*	***2528***
Official control—processing and trading	91 (6.74%)	11 (2.06%)			**102 (3.87%)**
*n analyzed*	*1350*	*533*	*627*	*124*	***2634***
Official control—Italian primary production			1 (0.29%)		**1 (0.20%)**
*n analyzed*	*149*	*1*	*346*		***496***
**Total n of n.c. samples**	**118 (4.37%)**	**18 (1.17%)**	**4 (0.39%)**	**2 (0.44%)**	**142 (2.43%)**
***Total n analyzed***	**2701**	**1536**	**1019**	**450**	**5854**

**Table 6:** Number of non-compliant (n.c.) samples for each element and the percentage of n.c. samples for each taxonomic group. In parentheses, the range of concentrations for each analyte (in mg/kg) detected in n.c. samples, taking into account measurement uncertainty, with the EU limits for each taxon/analyte combination listed below (in mg/kg).

Taxonomic Group	n.c. Samples (Range [Cd])	n.c. Samples [Pb]	n.c. Samples (Range [Hg])	Total n.c. Samples	% of n.c. Samples
Marine fish	0	0	**118** (0.74–13.00)	**118**	4.37%
*EU limit*	*0.05, 0.10, 0.30*	*0.30*	*0.5, 1.0*		
Cephalopods	**17** (1.40–5.60)	0	**1** (1.90)	**18**	1.17%
*EU limit*	*1.0*	*1.0*	*0.5*		
Bivalve mollusks	**3** (1.30–1.60)	**1** (3.20)	0	**4**	0.39%
*EU limit*	*1.0*	*1.5*	*0.5*		
Crustaceans	**2** (0.77–0.85)	0	0	**2**	0.44%
*EU limit*	*0.5*	*0.5*	*0.5*		
**Total**	**22**	**1**	**119**	**142**	**2.43%**

**Table 7:** Main parameters of total mercury concentrations obtained for different species groups of marine fish analyzed in this study. Concentrations values are expressed in mg/kg. Notes: * ML (maximum limit) of 1 mg/kg and ** ML of 0.5 mg/kg.

Total Mercury								
Species Group	n	Mean ± sd	Median	Max	p95	p99	n.c. Samples	% n.c. Samples
Swordfish *	522	0.75 ± 0.50	0.63	2.90	1.70	2.40	**59**	11.30%
Shark species *	247	0.75 ± 0.48	0.69	3.80	1.40	3.30	**16**	6.48%
Tuna species *	450	0.40 ± 0.40	0.30	3.00	1.10	1.90	**14**	3.11%
Others *	158	0.50 ± 1.06	0.37	13.00	1.06	2.10	**3**	1.90%
Others **	1142	0.13 ± 0.19	0.07	1.82	0.50	0.97	**26**	2.28%
**Total**	**2519**	**0.39 ± 0.51**	**0.23**	**13.00**	**1.20**	**1.90**	**118**	4.68%

**Table 8:** Estimated Weekly Intake (EWI) (µg/kg, body weight—b.w.—per week) of total mercury through the consumption of swordfish with mean and median values of [THg] obtained in this work for different population categories. Chronic swordfish consumption data for the Italian population (grams per kilogram of body weight per day) were obtained from two surveys included in the EFSA Comprehensive European Food Consumption Database.

Survey (Year)	Population Categories	Age Class (Years)	Mean Swordfish Consumption (g/kg b.w. per Week)	EWI THg (µg/kg b.w. per Week)	
				Mean [THg]	Median [THg]
Italian National Food Consumption Survey INRAN-SCAI (2005-06)	**All subjects** Adolescents	10–17	0.35	0.26	0.22
Italian National Food Consumption Survey INRAN-SCAI (2005-06)	**All subjects** Adults	18–64	0.28	0.21	0.18
Italian national dietary survey on adult population from 10 up to 74 years old (2018)	**All subjects** Adults	18–64	0.42	0.31	0.26
Italian National Food Consumption Survey INRAN-SCAI (2005-06)	**Consumers (n = 16; 6.5%)** Adolescents	10–17	5.95	4.46	3.75
Italian National Food Consumption Survey INRAN-SCAI (2005-06)	**Consumers (n = 106; 4.6%)** Adults	18–64	5.81	4.36	3.66
Italian national dietary survey on adult population from 10 up to 74 years old (2018)	**Consumers (n = 28; 3.9%)** Adults	18–64	10.64	7.98	5.03

## Data Availability

The original contributions presented in this study are included in the article. Further inquiries can be directed to the corresponding author.

## Supplementary Materials

The following supporting information can be downloaded at: https://www.mdpi.com/article/10.3390/foods14030451/s1, S1. Details of cadmium and mercury Maximum Levels (MLs) for different marine fish species set by the Commission Regulation (EU) 2023/915.
