# Supplementary material for: Monitoring of Cadmium, Lead, and Mercury Levels in Seafood Products: A Ten-Year Analysis
Source: Foods. 2025 Jan 30;14(3):451. doi: 10.3390/foods14030451 (PMC11817267; doi:10.3390/foods14030451)
Supplement: Supplementary file 1 [file foods-14-00451-s001.zip › foods-3416835-supplementary.pdf]

SUPPLEMENTARY MATERIAL S1

Details of cadmium and mercury Maximum Levels (MLs) for different marine fish species set by the Commission Regulation (EU) 2023/915.

CADMIUM

3.2.14.1 – ML: 0.05 mg/kg

Muscle meat of fish except species listed in 3.2.14.2, 3.2.14.3 and 3.2.14.4

3.2.14.2 – ML: 0.10 mg/kg

Muscle meat of the following fish:

Mackerel (*Scomber* species)

Tuna (*Thunnus* species, *Katsuwonus pelamis*, *Euthynnus species*)

Bichique (*Sicyopterus lagocephalus*)

3.2.14.3 – ML: 0.15 mg/kg

Muscle meat of bullet tuna (*Auxis* species)

3.2.14.4 – ML: 0.25 mg/kg

Muscle meat of the following fish:

Anchovy (*Engraulis* species)

Swordfish (*Xiphias gladius*)

Sardine (*Sardina pilchardus*)

MERCURY

3.3.1.1 ML: 0.50 mg/kg

Muscle meat of fish except species listed in 3.3.1.2 and 3.3.1.3

3.3.1.2 ML: 1.00 mg/kg

Muscle meat of following fish:

Axillary seabream (*Pagellus acarne*)

Black scabbardfish (*Aphanopus carbo*)

Blackspot seabream (*Pagellus bogaraveo*)

Bonito (*Sarda sarda*)

Common pandora (*Pagellus erythrinus*)

Escolar (*Lepidocybium flavobrunneum*)

Halibut (*Hippoglossus* species)

Kingklip (*Genypterus capensis*)

Marlin (*Makaira* species)

Megrim (*Lepidorhombus* species)

Oilfish (*Ruvettus pretiosus*)

Orange roughy (*Hoplostethus atlanticus*)

Pink cusk-eel (*Genypterus blacodes*)

Pike (*Esox* species)

Plain bonito (*Orcynopsis unicolor*)

Poor cod (*Trisopterus* species)

Red mullet (*Mullus barbatus barbatus*)

Roundnose grenadier (*Coryphaenoides rupestris*)

Sail fish (*Istiophorus* species)

Silver scabbardfish (*Lepidopus caudatus*)

Snake mackerel (*Gempylus serpens*)

Sturgeon (*Acipenser* species)

Surmullet (*Mullus surmuletus*)

Tuna (*Thunnus* species, *Euthynnus* species, *Katsuwonus pelamis*)

Shark (all species)

Swordfish (*Xiphias gladius*)

3.3.1.3 ML: 0.30 mg/kg

Muscle meat of the following fish:

Anchovy (*Engraulis* species)

Alaska pollock (*Theragra chalcogramma*)

Atlantic cod (*Gadus morhua*)

Atlantic herring (*Clupea harengus*)

Basa (*Pangasius bocourti*)

Carp (species belonging to the Cyprinidae family)

Common dab (*Limanda limanda*)

Mackerel (*Scomber* species)

European flounder (*Platichthys flesus*)

European plaice (*Pleuronectes platessa*)

European sprat (*Sprattus sprattus*)

Mekong giant catfish (*Pangasianodon gigas*)

Pollock (*Pollachius pollachius*)
